# Base Excision by Thymine DNA Glycosylase Mediates DNA-Directed Cytotoxicity of 5-Fluorouracil

**DOI:** 10.1371/journal.pbio.1000091

**Published:** 2009-04-28

**Authors:** Christophe Kunz, Frauke Focke, Yusuke Saito, David Schuermann, Teresa Lettieri, Jim Selfridge, Primo Schär

**Affiliations:** 1 Institute of Biochemistry and Genetics, Department of Biomedicine, University of Basel, Basel, Switzerland; 2 Institute of Cell Biology, ETH Zürich, Zürich, Switzerland; 3 Wellcome Trust Centre for Cell Biology, Institute of Cell and Molecular Biology, University of Edinburgh, Edinburgh, Scotland; Brandeis University, United States of America

## Abstract

5-Fluorouracil (5-FU), a chemotherapeutic drug commonly used in cancer treatment, imbalances nucleotide pools, thereby favoring misincorporation of uracil and 5-FU into genomic DNA. The processing of these bases by DNA repair activities was proposed to cause DNA-directed cytotoxicity, but the underlying mechanisms have not been resolved. In this study, we investigated a possible role of thymine DNA glycosylase (TDG), one of four mammalian uracil DNA glycosylases (UDGs), in the cellular response to 5-FU. Using genetic and biochemical tools, we found that inactivation of TDG significantly increases resistance of both mouse and human cancer cells towards 5-FU. We show that excision of DNA-incorporated 5-FU by TDG generates persistent DNA strand breaks, delays S-phase progression, and activates DNA damage signaling, and that the repair of 5-FU–induced DNA strand breaks is more efficient in the absence of TDG. Hence, excision of 5-FU by TDG, but not by other UDGs (UNG2 and SMUG1), prevents efficient downstream processing of the repair intermediate, thereby mediating DNA-directed cytotoxicity. The status of TDG expression in a cancer is therefore likely to determine its response to 5-FU–based chemotherapy.

## Author Summary

5-Fluorouracil (5-FU) has been used in clinical cancer therapy for more than four decades. Despite a moderate response rate and a high propensity of tumors to develop resistance to the drug, 5-FU remains a mainstay in the first-line treatment of colorectal cancer in particular. But precisely how 5-FU kills cancerous cells is not well understood. It is known, for example, that 5-FU affects RNA or DNA metabolism. Its DNA-directed cytotoxicity is thought to be based on extensive misincorporation of uracil and 5-FU into cellular DNA, and it has been proposed that the excision of these bases by uracil DNA glycosylases (UDGs) results in destructive DNA fragmentation, which can ultimately lead to cell death. However, the UDG responsible has not been identified. We now show that inactivation of only one of four mammalian UDGs, the thymine DNA glycosylase (TDG) in mouse and human cells is sufficient to confer resistance to 5-FU, whereas overexpression of TDG sensitizes cells to the drug. We provide further experimental evidence to show that excision of 5-FU from DNA by TDG, but not by other UDGs, inhibits efficient downstream processing of the lesion. This leads to an accumulation of DNA repair intermediates, which induce DNA damage signaling and, eventually, cell death. Thus, TDG activity in cells represents an important determinant of the DNA-directed cytotoxicity of 5-FU, an observation that might help us to understand the variable response to 5-FU treatments in cancer.

## Abstract 3

Targeted disruption of thymine DNA glycosylase (TDG) in mouse cells and down-regulation in human cancer cells establishes an important role of this protein in the cellular response to the anticancer drug 5-fluorouracil.

## Introduction

The antimetabolite 5-fluorouracil (5-FU) is an analog of uracil with a fluorine substitution at the C_5_ position. Developed as an inhibitor of thymidylate synthase (TS) [1], it has become an important compound in the first-line treatment of a range of human cancers, most prominently colorectal carcinomas [2]. Inside cells, 5-FU is converted to different active metabolites, including fluorodeoxyuridine monophosphate (FdUMP), fluorodeoxyuridine triphosphate (FdUTP), and fluorouridine triphosphate (FUTP) [2]. These metabolites have been implicated in both global RNA metabolism due to incorporation of the ribonucleotide FUMP into RNA, and DNA metabolism due to TS inhibition or direct incorporation of FdUMP into DNA. The therapeutic importance of its DNA-directed action is emphasized by a direct correlation of TS activity with the response rate of tumors or cancer cell lines to the treatment with 5-FU [3–5]. TS converts deoxyuridine monophosphate (dUMP) to deoxythymidine monophosphate (dTMP). FdUMP inactivates TS irreversibly upon docking to its nucleotide binding site and forming a stable complex with the cofactor 5,10-methylenetetrahydrofolate [6,7]. Thus, TS inhibition deprives the cell of the capacity to synthesize dTMP from dUMP and, thereby, elevates deoxyuridine triphosphate (dUTP) levels at the expense of deoxythymidine triphosphate (dTTP). The resulting dUTP/dTTP imbalance then favors the misincorporation of dUMP during DNA replication, giving rise to a dose-dependent increase in the steady-state level of DNA uracil [8,9].

It has been argued that the therapeutic effects of TS inhibition are based on the fragmentation of genomic DNA as a result of massive uracil excision by the replication-associated uracil DNA glycosylase (UDG) UNG2 connected with futile cycles of base excision repair (BER) [2,10,11]. Although UNG2 expression may be affected in human cells treated with fluorodeoxyuridine [12], such a scenario is not entirely consistent with other available experimental evidence. UNG2 clearly constitutes a major activity against the accumulation of uracil in genomic DNA [13], but its expression status does not affect the cellular resistance towards TS inhibition [14] and, hence, the survival of 5-FU–treated cells [15]. Thus, uracil excision by UNG2 is not likely to account for the DNA-directed cytotoxicity of 5-FU. In light of a recent report, however, showing that FdUMP gets itself incorporated into genomic DNA in 5-FU–treated cells, with levels even exceeding those of misincorporated uracil [9], it can be argued that the 5-FU rather than the uracil in the DNA is the cell toxic lesion.

Although UNG is the most efficient and specific UDG present in mammalian cells, it is not the only one. Single-strand–selective monofunctional uracil-DNA glycosylase 1 (Smug1) [16], thymine DNA glycosylase (TDG) [17], and methyl-CpG binding domain protein 4 (MBD4) [18,19] represent additional activities. All these enzymes are capable of processing uracil, as well as 5-FU, in DNA, albeit with different kinetic properties. Smug1 was shown to provide resistance to 5-FU–exposed cells [9], whereas MBD4 may contribute to the toxicity of the drug, arguably through DNA damage signaling [20]. Human TDG, originally discovered as a G•T mismatch-specific thymine DNA glycosylase [21], processes a broad range of substrates, including uracil and 5-FU. Although it has a strong preference for bases mispaired with a guanine, TDG excises 5-FU with a high efficiency, irrespective of whether the opposite base is a guanine or an adenine [17,22,23]. Consistently, plasmid-based in vitro repair assays with cell lysates have revealed a significant contribution of TDG to 5-FU excision [24].

Due to the redundancy of UDG activities that can contribute to 5-FU processing in cells, it is difficult to predict in which way and to what extent one or the other contributes to the cellular response to 5-FU, and thus to the efficacy of cancer therapies including 5-FU. Moreover, recent evidence from in vitro repair studies has implicated the postreplicative mismatch repair (MMR) system in the processing of 5-FU•G base pairs [24]. In part, this accounts for the increased resistance of MMR-deficient cells to treatment with fluoropyrimidines [25–27]. Given the general nucleotide imbalance induced by TS inhibition, however, the MMR-dependent toxicity of 5-FU is best explained by excessive formation and repair of DNA mispairs during replication [26,28]. Hence, the DNA-directed effects of 5-FU may reflect two lines of responses: the excision of 5-FU or U from DNA (5-FU/U•A, 5-FU/U•G) mainly by UDGs, and the excision of mismatched nucleotides mainly by MMR.

The objective of this study was to clarify the role of TDG in this context. We examined cellular and molecular responses to 5-FU exposure of matched *Tdg-*proficient and -deficient mouse embryonic fibroblasts and stem cells, as well as human HeLa cells. We show that TDG, of all UDGs, is responsible for the accumulation of DNA strand breaks, a delay in S-phase progression, and a persistent activation of DNA damage signaling upon treatment of cells with 5-FU, and that inactivation of *Tdg* by mutation causes resistance towards the drug. We conclude that TDG, unlike UNG2 and Smug1, mediates the DNA-directed cytotoxic effects of 5-FU.

## Results

### TDG Deficiency Confers Resistance towards 5-FU

**Figure 1:**
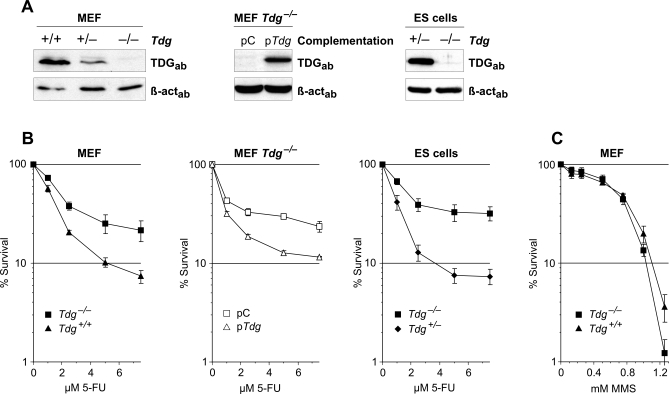
TDG-Deficient Mouse Cells Are Resistant to 5-FU (A) Western blot analysis of whole-cell protein extracts derived from SV40 immortalized MEFs (left), spontaneously immortalized MEFs (middle), and ES cell lines (right) used. *Tdg* genotypes are as indicated. A highly specific polyclonal anti-mouse TDG antibody (TDG_ab_) was used to detect TDG; beta-actin staining served as loading control (β-act_ab_). TDG is undetectable in extracts from *Tdg^−/−^* cells and reduced in heterozygous MEFs. Stable transfection of a *Tdg* expression construct (p*Tdg*) restores TDG levels in knockout MEFs. (B) TDG-deficient MEF and ES cells exhibit increased 5-FU resistance, and ectopic expression of wild-type *Tdg* in knockout MEFs restores 5-FU sensitivity. The sensitivity to increasing amounts of 5-FU was measured for the different cell lines after a continuous treatment of 48 h. Shown are survival curves as percentages of mock-treated cells. (C) TDG-deficient MEFs are not generally resistant to induced DNA base damage. Sensitivity to MMS was measured after a treatment for 1 h with increasing concentrations of MMS. Shown are survival curves as percentages of mock-treated cells. Shown are means ± standard error of the mean (SEM) from at least three independent experiments. pC, vector control; p*Tdg*, *Tdg*-expressing vector.

**Figure 2:**
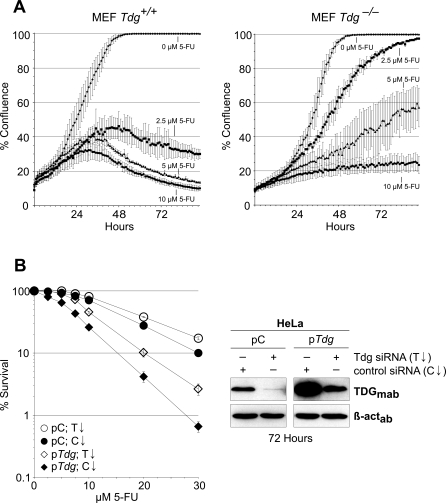
TDG-Induced Cell Death upon 5-FU Treatment (A) Exposure to 5-FU results in death of TDG-proficient cells (*Tdg^+/+^*), but slow growth of TDG-deficient (*Tdg^−/−^*) cells. The growth of TDG wild-type and knockout MEF cultures was monitored and recorded in real time during treatment with indicated concentrations of 5-FU. Shown are mean percentages of confluence at the respective time points with error bars representing standard deviations (SD) of three independent cultures. (B) HeLa cells overexpressing TDG show increased sensitivity towards 5-FU, whereas siRNA-mediated TDG knockdown results in increased 5-FU resistance. Shown is the clonogenic survival of HeLa cells with different TDG expression levels following treatment with 5-FU for 72 h (left). Data points represent percentages of colony-forming units (5-FU/mock; mean ± SEM) from at least three independent experiments. Corresponding TDG protein levels 72 h after plating of the cells are shown in the right panel. TDG was detected with a monoclonal anti-human TDG antibody (TDG_mab_) in extracts of cells treated with siRNA directed against TDG, or a corresponding siRNA control. pC, vector control; p*Tdg*, *Tdg* expressing vector; T↓, *Tdg* siRNA; C↓, control siRNA.

To investigate the role of TDG during 5-FU treatment of cells in culture, we established SV40 immortalized mouse embryonic fibroblasts (MEFs) with homozygous or heterozygous *Tdg* disruptions (*Tdg^+/+^*, *Tdg^+/−^*, *Tdg^−/−^*) from embryos (embryonic day [ED] 9.5) of heterozygous matings. The *Tdg* knockout allele, generated by classical gene targeting, had a replacement of exons 6 and 7, encoding parts of the catalytic core of TDG, with a neomycin resistance cassette (Figure S1). We then used litter-matched MEF lines for phenotypic examination. Western blotting with a polyclonal anti-mouse TDG antibody confirmed that neither full-length nor truncated versions of TDG were present in whole-cell extracts of the homozygous knockout MEFs, whereas heterozygous cells produced about half endogenous levels of the protein (Figure 1A; unpublished data). Continuous exposure of these MEFs to 5-FU for 48 h reduced living cell counts in a dose-dependent manner. However, compared to wild-type MEFs, TDG-deficient cells displayed a remarkable resistance (Figure 1B), and heterozygous cells showed an intermediate 5-FU sensitivity (Figure S2). These findings implicated a rate-limiting contribution of TDG to 5-FU–mediated cytotoxicity. A differential response of TDG-proficient and -deficient MEFs to 5-FU treatment was also observed in a real-time assessment of growth behavior. Whereas the cell numbers in cultures of TDG-proficient MEFs started to decline after 36 h of 5-FU exposure, TDG-deficient cells responded with a dose-dependent growth retardation only (Figure 2A).

Since immortalization by the SV40 large-T antigen (LTA) occurs through inactivation of antiproliferative proteins such as p53 or pRb and, thus, can affect the cellular DNA damage response [29,30], we also included spontaneously immortalized MEF cell lines in our analysis. To this end, we set up isogenic *Tdg*-proficient and -deficient MEF lines by stable transfection of a single clone with either a complementing *Tdg* transgene under the control of an SV40 promoter or the corresponding expression vector only (Figure 1A). Survival tests then showed that *Tdg* expression sensitized the *Tdg*-deficient cells to 5-FU to a level observed with the *Tdg*-proficient MEFs (Figure 1B). This confirmed that the 5-FU resistance of *Tdg* knockout cells is a direct consequence of the loss of TDG rather than of unspecific effects by SV40 LTA immortalization or other differences in clonal backgrounds.

To validate the resistance phenotype in cells that are naturally immortal, we examined the 5-FU response of *Tdg^+/−^* and *Tdg^−/−^* mouse embryonic stem (ES) cells generated in our laboratory (Figure 1A) (Y. Saito, unpublished data). Also there, the loss of TDG was associated with a remarkable increase in resistance towards 5-FU (Figure 1B). Hence, the mechanism by which TDG mediates cytotoxicity of 5-FU is active in very divergent cell types, including immortalized differentiated cells as well as undifferentiated stem cells.

To examine the drug specificity of this phenotype, we assessed the sensitivities of TDG-proficient and -deficient MEFs towards the monofunctional DNA alkylating agent methyl methansulfonate (MMS) (Figure 1C). At MMS concentrations yielding ≥10% cell survival, the TDG status did not significantly affect cellular sensitivity. At higher concentrations (≤5% survival), however, TDG-deficient cells were slightly more sensitive. Thus, TDG may contribute to the repair of MMS-induced DNA lesions, but it does not mediate cytotoxicity as it does in the case of 5-FU.

Finally, to address the impact of TDG on long-term survival of 5-FU–treated human cancer cells, we performed clonogenic survival assays. We established HeLa cell clones either stably transfected with a construct overexpressing human TDG from a cytomegalovirus (CMV) promoter or with the corresponding vector only. In addition, we applied a small interfering RNA (siRNA) approach to knockdown TDG in both HeLa cell clones. Continuous exposure of these HeLa cells to 5-FU for 72 h reduced colony forming units in a dose-dependent manner. The cellular sensitivity, however, significantly correlated with TDG protein levels (Figure 2B), being highest for TDG-overexpressing cells and lowest for the vector control cells with additional TDG knockdown. Thus, reduced TDG expression confers a long-term survival benefit to HeLa cells, establishing that the TDG expression status in a human cancer cell line determines its response to treatment with 5-FU.

### TDG Contributes to A•5-FU Repair in Nuclear Extracts

**Figure 3:**
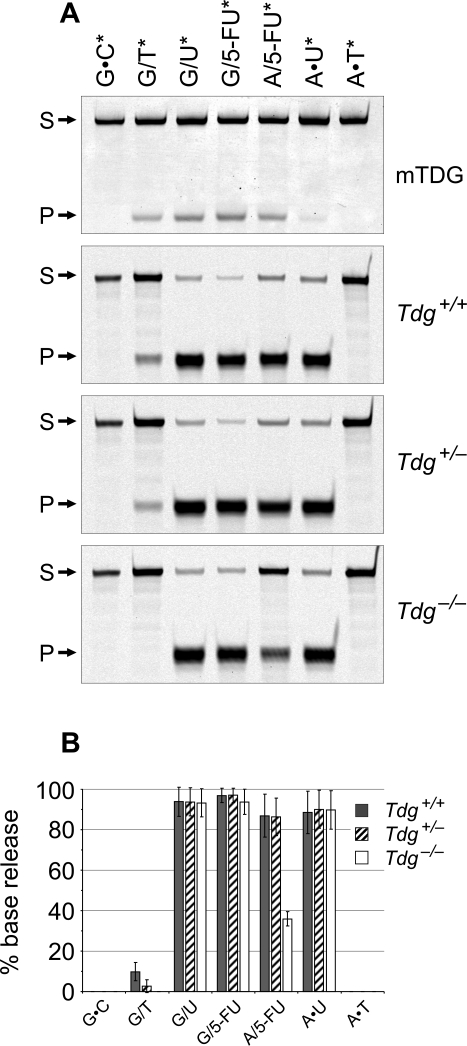
Involvement of TDG in Processing of Uracil and 5-FU (A) Base release activities of purified recombinant mouse TDG (mTDG) and nuclear protein extracts of TDG wild-type (*Tdg^+/+^*), heterozygous (*Tdg^+/−^*), and knockout (*Tdg^−/−^*) MEFs on uracil, 5-FU, and G•T containing synthetic 60-mer DNA duplexes. Shown are representative results of base release assays with the intact substrate DNA strands (S) and the cleaved products (P) resolved on denaturing polyacrylamide gels. All reactions were performed in the presence of the UNG inhibitory UGI peptide. Purified TDG processes thymine, uracil, and 5-FU when opposite guanine as well as 5-FU paired with adenine, but only inefficiently uracil opposite adenine. (B) Quantitation of base release activities in nuclear extracts. G•T processing activity is reduced in protein extracts of heterozygous cells and absent from knockout extracts. *Tdg* knockout extracts also show a significant reduction of A•5-FU processing. All other uracil- and 5-FU–containing substrates were processed with similar efficiencies by all three nuclear extracts. Data are presented as means ± SD from three independent experiments. An asterisk (*) indicates the 5'-fluorescein-labeled strand. doi:10.1371/journal.pbio.1000091.g003

Biochemical studies revealed that the human TDG acts on a rather broad range of substrates, including G•U and G•5-FU mispairs, but also 5-FU base-paired with adenine. 5-FU, in fact, turned out to be the only base that is efficiently processed by TDG in the normal base-pairing configuration or even in single-stranded DNA [22,23]. We thus reasoned that the excision of 5-FU and/or uracil from genomic DNA by TDG might be a source of 5-FU–mediated cytotoxicity in TDG-proficient cells. To test this, we first validated the 5-FU– and uracil-processing abilities of purified mouse TDG in base-release assays. This showed that, like its human counterpart, the mouse protein excises thymine, uracil, and 5-FU opposite guanine, but also 5-FU paired with adenine, all with comparable efficiencies (Figure 3A). A•U containing homoduplex DNA, however, was hardly processed, suggesting that TDG does not contribute significantly to the repair of A•U base pairs.

To assess the contribution of the endogenous mouse TDG to overall uracil and 5-FU processing, we then analyzed the activities present in nuclear extracts of *Tdg* wild-type, heterozygous, and null mutant MEFs. G•T processing served as a control and was detectable in extracts from wild-type, but not from homozygous *Tdg* knockout cells (Figure 3A). Compared to homozygous wild-type cells, heterozygous *Tdg* knockout cells showed reduced thymine excision activity, consistent with the reduced levels of TDG in these cells (Figures 3B and 1A). Thus, TDG constitutes the major and rate-limiting mismatch-specific thymine excision activity in these cells, suggesting that MBD4, another G•T processing DNA glycosylase [31,32], is not or only poorly active. A lack of MBD4 activity was observed also with protein extracts from mouse ES cells (Y. Saito, unpublished data) and in previous studies with different cell systems [24,33]. Considerably higher amounts of nicked DNA products were detected for all uracil- and 5-FU–containing substrates. Removal of uracil and 5-FU from G•U, G•5-FU, and A•U was equally efficient irrespective of the TDG status. Excision of 5-FU from an A•5-FU substrate, however, was significantly reduced in extracts from *Tdg^−/−^* MEFs (Figure 3A and 3B). The remaining activity on A•5-FU, but also the efficient processing of A•U, G•U, and G•FU in extracts from *Tdg* knockout MEFs most likely reflects the action of other UDGs with overlapping substrate spectra. As the highly efficient UNG2 was inhibited by addition of saturating amounts of UGI peptide in these assays (unpublished data) and MBD4 activity was not detected, Smug1 most likely represents the redundant uracil- and 5-FU–processing activity observed here. The data therefore suggest that, under conditions of low UNG2 activity in cells, such as outside of S-phase when UNG2 is down-regulated [33–35], TDG constitutes a major and rate-limiting A•5-FU–processing activity.

This is consistent with measurements of 5-FU incorporation into genomic DNA following 5-FU treatment. The genomic levels of uracil and 5-FU upon treatment with 10 μM 5-FU for 48 h were four and 11 times higher in TDG knockout MEFs (3.4 × 10^5^ U residues; 1.7 × 10^6^ 5-FU residues) than in wild-type cells (8.2 × 10^4^ U residues; 1.5 × 10^5^ 5-FU residues), respectively, and this compares to the levels measured in 5-FU–treated *Smug1 knockdown* cells (D. Barnes, personal correspondence; [9]). Not only do these data confirm that 5-FdUMP gets incorporated into genomic DNA, they also establish that both TDG and Smug1 constitute the major activities processing these lesions, whereas UNG2 does not appear to contribute significantly as also implicated by uracil/5-FU incorporation measurements and sensitivity tests with *Ung^−/−^* knockout cells [9,15].

### 5-FU Treatment Induces TDG-Dependent DNA Strand Breaks

**Figure 4:**
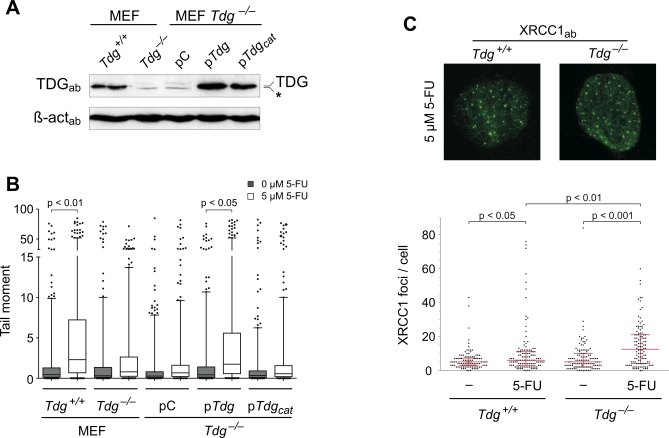
5-FU–Induced DNA Strand Breaks Are Reduced in TDG-Deficient Cells whereas Overall Repair Activity Is Increased. (A) Complementation of *Tdg* knockout MEFs with wild-type and catalytically deficient TDG. Stable transfectants of *Tdg^−/−^* MEFs ectopically expressing either TDG variant from the native promoter show TDG levels about the same as endogenous, as detected by western blotting. (B) Reduced levels of 5-FU–induced DNA strand breaks in cells lacking active TDG. Steady-state levels of DNA single- and double-strand breaks in the cell lines indicated were assessed by the alkaline Comet assay using automated comet tail moment analysis. 5-FU treatment results in a significant tail moment increase in wild-type, but not in *Tdg* knockout MEFs. The generation of 5-FU–specific DNA strand breaks in *Tdg* knockout cells is restored by complementation with wild-type *Tdg*, but not with the catalytically inactive mutant. Shown are box plots with individual tail moments per cell, medians, interquartile ranges (boxes), 2.5%–97.5% percentiles (whiskers) and outliers (dots) of pooled data (600 to 900 cells) obtained from three independent experiments. (C) 5-FU treatment triggers DNA SSB repair in TDG wild-type and knockout cells. The top panel shows nuclei of Tdg-proficient and -deficient cells stained with a polyclonal anti-XRCC1 antibody (XRCC1_ab_) after 5-FU treatment. The statistical analysis of XRCC1 foci per cell across the populations analyzed (*n* ≥ 100 cells per population) is shown as a scatter plot with medians and the interquartile ranges. pC, empty vector; p*Tdg*, vector expressing TDG; p*Tdg_cat_*, vector expressing a catalytic dead variant.

5-FU treatment has been associated with the generation of DNA strand breaks [2,10,11]. In the light of our biochemical evidence, implicating TDG in processing genomic A•5-FU base pairs, this might be accounted for by an accumulation of apyrimidinic/apurinic sites (AP-sites) in DNA. Through further processing by the BER system, spontaneous breakage, or stalling of DNA polymerases, these could give rise to increased levels of single-stranded DNA breaks (SSBs) in cells. To test this hypothesis, we applied alkaline Comet analyses to assess 5-FU–induced SSB formation in TDG-proficient and -deficient MEFs, as well as in complemented knockout cells stably expressing an ectopic wild-type or catalytic mutant *Tdg* (Figure 4A). To avoid *Tdg* overexpression artifacts in the latter [33], we made use of constructs that drive *Tdg* expression from its endogenous promoter (D. Schuermann, unpublished results). Automated analyses of Comet tail moments then showed similar background levels of DNA strand breaks in all untreated cell populations. Following treatment with 5 μM 5-FU for 24 h and a recovery of another 24 h, however, the tail moments increased significantly above background in TDG-proficient populations, whereas no significant increase was detected for TDG-deficient cells (Figure 4B). Remarkably, cells complemented with the catalytic-inactive mutant form of TDG did not show significantly elevated tail moments after 5-FU treatment. These data indicate that base excision by TDG accounts for the increase in steady-state levels of DNA strand breaks observed upon treatment of cells with 5-FU.

DNA processing after base excision generates SSBs, feeding into a SSB repair pathway whereby XRCC1 plays a central role [36]. To address a possible engagement of SSB repair following 5-FU treatment, we quantified nuclear XRCC1 foci by immunofluorescence detection of the endogenous protein with specific mono- and polyclonal antibodies [37]. This showed indeed that the median number of XRCC1 foci per cell increased after 24 h of low-dose 5-FU treatment and a recovery of 24 h in the absence of the drug (Figure 4C). We therefore conclude that uracil/5-FU excision from genomic DNA activates SSB repair processes at the site of the lesion. Remarkably, 5-FU treatment induced significantly more XRCC1 foci in *Tdg* knockout cells, indicating higher SSB repair activity in these cells, most probably downstream of uracil/5-FU excision by the remaining UDG activities. Thus, in wild-type cells TDG may compete with these glycosylases for the 5-FU substrates, generate AP-sites, but then prevent efficient downstream processing of the repair intermediates by the SSB repair pathway.

### 5-FU Arrests MEFs in S-Phase and Activates DNA Damage Responses

**Figure 5:**
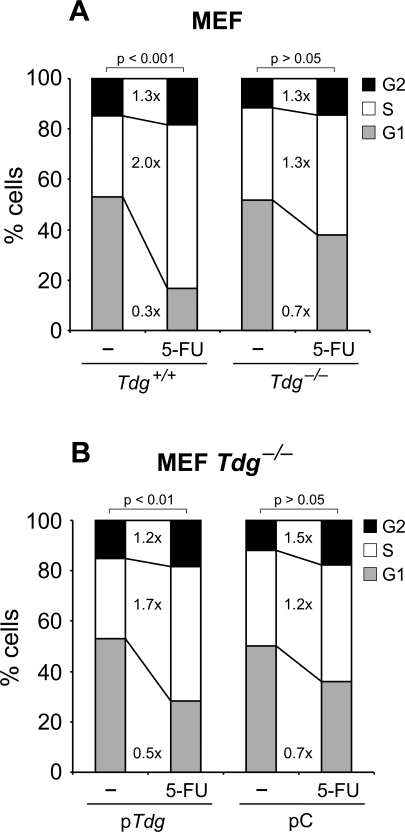
5-FU Treatment Induces a TDG-Dependent S-Phase Delay The histograms show the effect of the 5-FU treatment on the relative cell-cycle distribution (% cells) of TDG-proficient and -deficient MEFs (A), and of TDG knockout cell lines stably transfected with a plasmid expressing *Tdg* from its authentic promoter (B). 5-FU treatment of TDG-proficient cells results in a significant accumulation cells in S-phase at the expense of G1 cells, whereas TDG-deficient cells show only insignificant changes in cell-cycle distribution. Expression of wild-type *Tdg* in knockout MEFs partially restored the 5-FU–dependent S-phase delay. The data shown represent averages of three independent experiments with fold changes upon 5-FU treatment. pC, empty vector; p*Tdg*, vector expressing TDG.

5-FU treatment was shown to delay or even arrest S-phase progression in HeLa and DT40 cells [38,39]. To address the role of TDG in this context, we determined the cell-cycle profiles of TDG-proficient and -deficient MEFs following 5-FU treatment. Relative to the mock control, treatment for 24 h with 5 μM 5-FU and subsequent cultivation in drug-free medium for additional 24 h resulted in a significant enrichment of *Tdg* wild-type cells in the S (2-fold) and G2/M phases (1.3-fold) of the cell cycle (Figure 5A). This enrichment occurred at the expense of the G1 cell population, which was reduced by a factor of three. By contrast, the 5-FU–induced changes in cell-cycle distribution of TDG-deficient MEFs were less pronounced and not statistically significant (Figure 5A). Since a treatment with hydroxyurea (HU) impeded S-phase progression equally in both cell lines (unpublished data), a defective intra–S-phase DNA-damage checkpoint in the TDG knockout MEFs can be excluded. To corroborate the TDG dependence of the 5-FU–mediated S-phase delay, we compared the response of *Tdg* knockout cells complemented by stable expression of endogenous levels of wild-type *Tdg* with that of a vector control. Also in this setting, the S-phase delay induced by 5-FU treatment was significantly more pronounced in the *Tdg*-expressing cell line (1.7-fold vs. 1.2-fold) (Figure 5B). Thus, TDG contributes to cell-cycle responses following 5-FU treatment.

**Figure 6:**
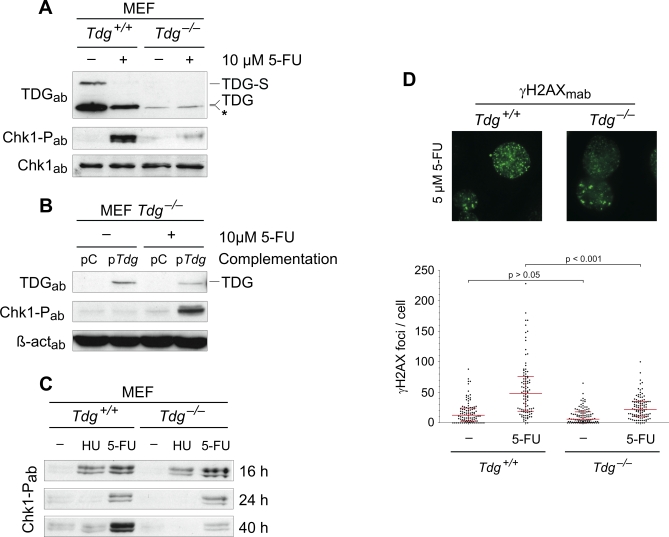
TDG-Dependent Activation of DNA Damage Responses upon 5-FU Treatment (A–C) TDG mediates late Chk1 activation following 5-FU treatment. Activation of Chk1 in TDG-proficient and -deficient MEFs (A) as well as in complemented knockout cells (B) was determined by western blotting with a S345 phospho-specific antibody against Chk1 (Chk1-P_ab_). After treatment with 10 μM 5-FU, wild-type but not TDG-deficient MEFs show a strong accumulation of S345 phosphorylated Chk1. Total Chk1 protein is the same in both MEF lines before and after 5-FU treatment (Chk1_ab_). TDG levels in wild-type cells, detected with a specific anti-mTDG antibody (TDG_ab_), are reduced in 5-FU–exposed cells, reflecting an accumulation of cells in S-phase, where TDG is absent. *Tdg* knockout MEFs stably expressing an ectopic copy of *Tdg* (B) contain low levels of TDG, which is sufficient to induce Chk1 activation upon 5-FU treatment. (C) Dynamics of Chk1 activation in TDG-proficient and -deficient MEFs during and after exposure to 5-FU or HU. The 5-FU–containing (10 μM) or HU-containing (2.5 mM) medium was replaced with drug-free medium after 24 or 16 h, respectively. Samples were taken at the time points indicated and analyzed for Chk1 S345 phosphorylation by western blotting. After 16 h into treatment, activated Chk1 appears equally in extracts from 5-FU– and HU-treated cells; at 24 h, the Chk1-p signal is undetectable in the HU-treated samples and significantly reduced in 5-FU–treated cells; at 40 h, significant levels of phosphorylated Chk1 reappear in 5-FU–exposed TDG-proficient MEFs but not in TDG-deficient MEFs. (D) The induction of γH2AX foci by 5-FU treatment is significantly reduced in TDG-deficient MEFs. The top panels show examples of MEFs immunostained with a monoclonal antibody against γH2AX (γH2AX_ mab_) after treatment with 5 μM 5-FU. The statistical analysis of γH2AX foci per cell across the populations analyzed (*n* > 95 cells per population) is depicted in the lower panel as scatter plot with medians and the interquartile ranges. pC, empty vector; p*Tdg*, vector expressing TDG; TDG-S, TDG modified with SUMO. An asterisk (*) indicates an unspecific cross-reaction of the secondary antibody

5-FU–induced cell-cycle arrest in early S-phase was shown previously to depend on Chk1, an effector protein kinase [38,39] that gets activated in response to DNA damage or replication stress through ATR-dependent serine (S317 and S345) phosphorylation (Chk1-p) [40]. We examined the role of TDG in checkpoint activation following treatment of cells with 10 μM 5-FU for 24 h and a recovery in drug-free medium for another 24 h. Immunoblotting of whole-cell extracts with a S345 phospho-specific Chk1 antibody confirmed significant activation of the kinase in *Tdg* wild-type cells (Figure 6A). In extracts of 5-FU–treated *Tdg* knockout cells, however, 5-FU–induced Chk1 phosphorylation was hardly detectable. This was not due to an absence of Chk1 or an inability to phosphorylate the kinase in these cells; immunodetection of total Chk1 protein confirmed similar levels in wild-type and knockout extracts (Figure 6A), and the replication inhibitor HU induced Chk1 phosphorylation in both cell types (Figure 6C). Note that the reduced TDG protein levels detected in the extracts of 5-FU treated wild-type MEFs (Figure 6A) reflect the accumulation of the cells in S-phase, where TDG is not expressed [33]. Finally, stable transfection of a TDG-expressing plasmid restored 5-FU–inducible Chk1 phosphorylation in the *Tdg* knockout cells (Figure 6B).

To address the dynamics of Chk1 activation, we monitored S345 phosphorylation during a 24-h treatment with 10 μM 5-FU and an additional recovery time of 24 h in the absence of 5-FU. Weak Chk1 phosphorylation became detectable after 16 h of treatment both in *Tdg* wild-type and knockout MEFs. This initial signal persisted throughout a treatment period of 24 h (Figure 6C), but declined gradually during the subsequent recovery period. Strikingly, however, in TDG-proficient cells, Chk1 phosphorylation reappeared at 40 h into the time course, which is 16 h after removal of the drug (Figure 6C). Thus, 5-FU elicits an early checkpoint response that is independent of TDG and a late response that depends on TDG.

Another readout of ATM- or ATR-dependent DNA-damage responses is the formation of nuclear foci containing a phosphorylated variant of histone H2AX [41]. γH2AX is considered a marker of DNA damage, including DNA double-strand breaks that may occur during DNA replication when moving forks encounter damage in the parental strands. We thus measured changes in the steady-state levels of γH2AX foci in TDG-proficient and -deficient MEF populations upon treatment with 5 μM 5-FU for 24 h and additional recovery for 24 h in drug-free medium. Although both mock-treated cell lines showed similar levels of γH2AX foci, the wild-type MEFs accumulated significantly higher numbers of γH2AX foci than the TDG-deficient cells during 5-FU treatment (Figure 6D). This corroborates that 5-FU treatment induces DNA strand breaks and, consequently, DNA-damage signaling in a TDG-dependent manner.

## Discussion

Despite many years of clinical application, the mode of action underlying the therapeutic efficacy of the antimetabolite 5-FU has remained elusive. Circumstantial evidence, however, has suggested that a significant part of its cancer-directed cytotoxicity is mediated through the excision of misincorporated uracil or 5-FU from genomic DNA, saturating the cellular SSB repair capacity [2]. Such a scenario clearly implicates a critical role for UDGs in mediating the cytotoxicity. However, Smug1 was reported to protect cells from the cytotoxic effects of 5-FU, and the status of UNG2, the catalytically most efficient UDG of all, does not seem to affect cellular sensitivity [9,14,15]. MBD4-deficient cells were shown to have a survival benefit on 5-FU [20], but this may not be linked to the loss of DNA glycosylase activity since immunodepletion of the enzyme did not alter the 5-FU repair capacity of nuclear extracts [24]. Thus, whether or not and to what extent UDG activities are responsible for the DNA directed 5-FU toxicity remained unclear.

Our data now establish a significant contribution of TDG to DNA-directed 5-FU cytotoxicity. We show that inactivation of *Tdg* in MEFs, but also in ES cells, results in a marked cellular resistance towards 5-FU, which can be complemented by expression of wild-type *Tdg*. The same inverse correlation between TDG proficiency and 5-FU sensitivity was observed with the human cancer cell line HeLa; TDG overexpression in HeLa cells increased cellular sensitivity to 5-FU treatment, whereas siRNA-mediated TDG knockdown in the same cells provided resistance. This phenotype is apparently specific to 5-FU as the TDG-deficient MEFs showed no resistance when treated with MMS. Recently, An et al. [9] demonstrated the accumulation of appreciable amounts of FdUMP in genomic DNA following 5-FU treatment of cells, and further presented genetic evidence consistent with 5-FU rather than uracil in DNA being the toxic lesion. Our data show that nuclear extracts from TDG-deficient cells excise 5-FU from A•5-FU base pairs with significantly reduced efficiency when compared to wild-type extracts. Although this difference is detectable only upon inhibition of the highly active UNG2 by the UGI peptide, it nevertheless implicates a rate-limiting contribution of TDG to the excision of 5-FU in non–S-phase cells, where UNG2 activity is down-regulated [33–35]. In agreement with this, we found TDG-deficient MEFs to accumulate significantly higher levels of 5-FU in their DNA than TDG-proficient cells following 5-FU exposure. Hence, TDG processes 5-FU in genomic DNA and may thus contribute to the cytotoxicity of the drug. Given the inability of TDG to excise U from the A•U base pair on the one hand [21,22], and the comparably high contribution of TDG to A•5-FU processing in nuclear extracts on the other hand, we argue that the primary TDG relevant cytotoxic DNA lesion is the A•5-FU base pair.

Consistent with a concept of DNA repair generating lethal DNA strand breaks upon 5-FU treatment [2], our Comet data show an increase of the tail moment in 5-FU–treated MEFs, and this effect is largely dependent on the presence of a catalytically active TDG. At the same time, we observed a significant increase of the number of XRCC1 foci per cell, suggesting that 5-FU treatment triggers DNA SSB repair [36]. Strikingly, after 5-FU treatment, *Tdg* knockout cells produced significantly higher levels of XRCC1 foci than their wild-type counterparts, suggesting that the loss of TDG enhances overall SSB repair activity while reducing lethal 5-FU processing.

Why then is the excision of 5-FU (or uracil) by TDG cytotoxic, whereas excision by other UDGs, particularly Smug1, protects against cell death [9]? The difference may relate to the distinct modes of action of these enzymes. Both TDG and Smug1 bind AP-sites in DNA, albeit with different affinities. The dissociation of the glycosylases from these repair intermediates is therefore rate limiting for further processing [23,42]. However, whereas Smug1 can be made to turnover in the presence of APE1, the downstream-acting endonuclease competing for the AP-site [42], efficient AP-site release (and stimulation by APE1) of TDG requires a SUMO modification–induced conformational change that reduces its DNA binding affinity [43,44]. Thus, base excision by Smug1 may connect to a straightforward downstream repair process, whereas base excision by TDG may be associated with delayed repair of the AP-site, possibly due to saturation of the SUMOylation system. Some AP-sites generated by TDG would thus escape repair until they eventually interfere with DNA replication, leading to fork stalling and collapse, and activation of replication stress or DNA-damage checkpoints [45]. Indeed, we and others found 5-FU treatment to affect the progression of cells through S-phase [39,46], and this effect was associated with activation of the Chk1 kinase that contributes to S and G2/M checkpoints [47]. Both an accumulation of cells in S-phase and the activation Chk1 upon 5-FU exposure were virtually absent in TDG-deficient MEFs, and were in line with reduced levels of 5-FU–induced DNA strand breaks and γH2AX foci in these cells.

**Figure 7:**
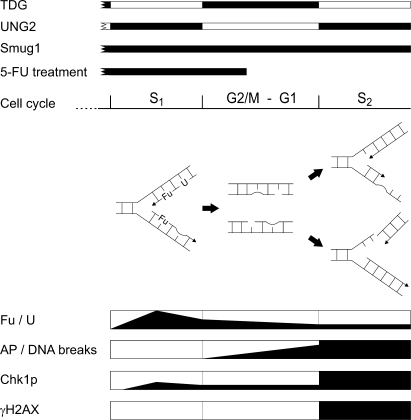
TDG-Dependent 5-FU Cytotoxicity Illustrated are the cell-cycle distributions of the three relevant UDGs, TDG, UNG2, and Smug1 (top), together with expected levels of genomic 5-FU, uracil, AP-sites, and the observed Chk1 activation following 5-FU treatment (bottom). TDG is present during the G2/M and G1 phases but is degraded prior to and absent from S-phase. UNG2 shows a strictly inverse regulation whereas Smug1 is expressed throughout the entire cell cycle. Treatment with 5-FU for 24 h gives rise to misincorporation of appreciable levels of 5-FU and uracil during S-phase (S1), resulting in Chk1 activation by ongoing replication-associated UNG2 and Smug1-dependent BER. Although Smug1 and UNG2 will initiate faithful repair of uracil and 5-FU bases directly after DNA synthesis, these pathways will become saturated under 5-FU exposure and, in addition, are relatively inefficient in processing the 5-FU•A base pairs. Hence, some of them will persist in the DNA into the subsequent G2 and G1 phases of the cell cycle. There, TDG will initiate repair, but turnover with a low rate, leading to an accumulation of AP-sites and/or DNA SSBs. During the subsequent S-phase, these repair intermediates will interfere with DNA replication, causing replication fork stalling, fork collapse, DNA double-strand breaks, and a second round of Chk1 activation. Due to genome fragmentation, cells will then induce apoptosis.

We reported previously that TDG is absent from S-phase cells due to programmed degradation by the proteasome system at the G1–S boundary [33]. This is consistent with the dynamics of Chk1 activation and cell death in our experiments, both indicating that the TDG-dependent cytotoxic action is temporally separated from the incorporation of 5-FU (and U) into DNA. On the basis of these findings, we can now put forward a model for how temporally separated 5-FU/uracil misincorporation and repair processes can determine the cellular responses to 5-FU (Figure 7). Upon exposure to 5-FU, 5-FU/uracil will be misincorporated into DNA during DNA replication in S-phase of the cell cycle. In this context, UNG2 will act efficiently on uracil (A•U and G•U) but less so on 5-FU [9], whereas Smug1 (and MBD4) may process the same lesions but with lower efficiencies. These repair events will activate the first wave of checkpoint responses that is TDG independent. Due to saturation of uracil repair, considerable amounts of A•5-FU base pairs will persist in the DNA into the subsequent phases of the cell cycle, where UNG2 is down-regulated, and they will be attacked mainly by TDG and Smug1. AP-sites generated by TDG will be protected from repair due to rate-limiting dissociation of the glycosylase and, hence, accumulate and interfere with the replication machinery in the subsequent S-phase. This will give rise to a second wave of checkpoint activation (Chk1 phosphorylation and formation of γH2AX), this time TDG dependent, which is correlated with the occurrence of DNA strand breaks, even if the cells are no longer cultivated in the presence of 5-FU.

Notably, according to a recent report, breast cancer patients carrying a specific polymorphism in XRCC1 have a significantly reduced risk of recurrence and show better long time survival following a combination therapy with cyclophosphamide–methotrexate–5-FU [48]. The same polymorphism was previously reported to reduce DNA repair activity of XRCC1 [48,49], suggesting that inactivation of DNA SSB repair can improve the efficacy of 5-FU treatment. The data presented here for TDG, and previously for Smug1 [9], are consistent with 5-FU excision being responsible for the generation of a significant fraction of AP-sites and DNA SSBs following 5-FU treatment. It is now becoming clear that the efficiency of coupling downstream repair with base excision, presumably through XRCC1, depends on the biochemical properties of the DNA glycosylase engaged and critically determines the cellular responses to the drug. It will therefore be important to examine to what extent the status of TDG activity correlates with the response of tumors to 5-FU–based chemotherapy.

## Materials and Methods

### Reagents, antibodies, and *Tdg* expression constructs.

Chemicals and reagents were purchased from Sigma, Complete protease inhibitor from Roche, RNase from Qiagen and UGI from New England Biolabs. LIF was from Chemicon-Millipore, sodium pyruvate from Invitrogen, and all other supplements or cell culture media from Sigma. The polyclonal rabbit anti-mTDG antiserum was newly generated by immunization with recombinant full-length mouse TDGa (Primm Labs). Rabbit anti-Chk1 (#2345) and rabbit anti-Chk1-Ser345p (#2341) antibodies were from Cell Signaling, the mouse anti–β-actin (ab8226) antibody was from Abcam, the rabbit anti-XRCC1 (X0629) was from Sigma, and the mouse anti-γH2AX (#05–636) was from Chemicon-Millipore. The monoclonal rat anti-hsTDG antibody was described in [44]. The secondary horseradish peroxidase–conjugated antibodies against mouse (NXA931) or rabbit (NA934V) were purchased from GE Healthcare Life Sciences, the secondary Cy2-conjugated donkey anti-mouse IgG (715-225-151) and anti-rabbit IgG (711-225-152) antibodies, and donkey serum (017-000-001) were from Jackson ImmunoResearch Laboratories, and the horseradish peroxidase–conjugated antibody against rat (A5795) was from Sigma. ON-TARGETplus SMARTpool siRNA against human Tdg (L-003780–01), ON-TARGETplus Non-targeting Pool control siRNA (D-001810–10) and DharmaFECT siRNA transfection reagent (T-2001) were from Thermo Scientific Dharmacon.

For bacterial expression of an N-terminally 6xHis-tagged mouse TDG, the murine *TdgA* cDNA (GenBank accession number: NM_172552) was cloned into pET28c (Novagen-Merck). Mammalian expression constructs were obtained by PCR cloning of the mouse *TdgA* sequence into pSG5-HH25 [44] or pTCO4 (D. Schuermann, unpublished data) for expression controlled by the SV40 or the authentic *Tdg* promoter, respectively. Constructs expressing human Tdg from a CMV promoter were obtained by PCR cloning of the *Tdg* cDNA (GenBank: NM_003211) into pCEP4 (Invitrogen). In vitro mutagenesis of mouse *Tdg* was performed using the QuikChange site-directed mutagenesis kit (Stratagene). PCR primer sequences and vector maps are available on request.

### Cell culturing.

For spontaneous immortalization, primary cells from *Tdg^−/−^* embryos (ED 9.5) were expanded in growth medium (DMEM, 10% FCS, 2 mM L-glutamine). Cultures were kept at <90% confluency at all times. Spontaneously immortalized cell lines were obtained through prolonged cultivation in medium containing 20% FCS and stored in growth medium containing 10% DMSO at 5 × 10^6^ cells/ml in liquid nitrogen. Alternatively, primary cells were immortalized by transfection with a plasmid expressing SV40 large T-antigen. Immortal MEFs were cultivated in growth medium containing penicillin/streptomycin (pen/strep) at 37 °C with 5% CO_2_. For complementation, *Tdg^−/−^* cell lines were transfected at 70% confluency with 1 μg of plasmid DNA and the Transfectin reagent (BioRad). Puromycin-resistant cells were selected and further maintained in medium supplemented with 1.5 μg/ml puromycin. For protein extraction, Comet assays or FACS analysis, 1 × 10^6^ (mock) or 2 × 10^6^ (5-FU) cells were seeded into 10-cm culture dishes and incubated for 24 h. Cells were treated with indicated 5-FU concentrations for 24 h and washed with PBS. After additional incubation for 24 h in drug-free medium, cells were harvested by trypsinization. Mouse ES cells with a homozygous disruption of the *Tdg* gene were selected with increasing concentrations of neomycin from ES cells heterozygous for *Tdg*. ES cells were passaged in ES medium (DMEM, 15% heat-inactivated FCS, 2 mM L-glutamine, 0.1 mM β-mercaptoethanol, 1 mM sodium pyruvate, 1× nonessential amino acids, 1× pen/strep, 1,000 U/ml LIF) in the presence of γ-ray–inactivated feeder cells, which were removed prior to sensitivity assays. HeLa cells were maintained in growth medium containing penicillin/streptomycin (pen/strep) at 37 °C with 5% CO_2_.

### Protein extraction and western blotting.

Cells were washed with ice-cold PBS and lysed for 30 min on ice in lysis buffer (50 mM Na-phosphate [pH 8.0], 125 mM NaCl, 1% NP-40, 0.5 mM EDTA, 1 mM PMSF, 1 mM DTT, 1× Complete protease inhibitors, 2× phosphatase inhibitor cocktail 1 and 2). Extracts were clarified by centrifugation (15 min, 20,000*g*, 4 °C). Protein concentrations were determined using the Bradford reagent (BioRad). A total of 50 μg of soluble protein was separated in 10% SDS-polyacrylamide gels and transferred to a nitrocellulose membrane (Millipore). Membranes were washed once with TBS-T (100 mM Tris/HCl [pH 8.0], 150 mM NaCl, 0.1% Tween20) and incubated with blocking buffer (TBS-T, 5% dry milk) for 1 h at room temperature (RT). Blocked membranes were washed once with TBS-T for 5 min before incubation with the primary antibody for 1 h at 33 °C (anti-mTDG) or RT (anti–β-actin) in blocking buffer or overnight at 4 °C in TBS-T containing 5% BSA (anti-Chk1 and anti-Chk1-Ser345p). Dilutions were: 1:10,000 for the rabbit anti-mTDG antibody and the mouse anti-β-actin, 1:1,000 for the rabbit anti-Chk1 and the rabbit anti-Chk1-Ser345p. The washing steps after hybridization were: once at 33 °C and twice at RT for 15 min (anti-mTDG), three times at RT for 10 min (anti-β-actin), and three times for 5 min at RT (anti-Chk1 and anti-Chk1-Ser345p). Both secondary horseradish peroxidase–conjugated antibodies were diluted 1:5,000 in blocking buffer and hybridized to the membranes for 1 h at RT. After three washing steps of 10 min at RT, detection of the signals was carried out using the Immobilon Western Chemiluminescent HRP Substrate (Millipore).

### Cell-sensitivity assays.

Cell viability of MEFs and ES cells was measured by the Cell Counting Kit-8 (Dojindo). Triplicate cultures of each cell line were plated in 96-well plates at 1 × 10^3^ cells per well and pre-incubated in the respective growth medium. 5-FU or MMS was added to final concentrations as indicated. Cells were exposed for 48 h to 5-FU (MMS: 1 h, and an additional 47 h in normal growth medium), and then washed with PBS before incubation in medium containing the WST-8 substrate at 37 °C. After incubation for 2 h (MEF) or 4 h (ES), the cell density was measured indirectly by quantification of the solubilized formazan product at 450 nm with a SpectraMax340 microplate spectrophotometer (Molecular Devices). For real-time analysis of cell growth during 5-FU treatment, MEF cells were seeded in 12-well plates at approximately 5% confluence. Following a preincubation of 24 h, the growth medium was exchanged with medium containing 5-FU concentrations as indicated. Cell proliferation was recorded with an automated IncuCyte microscope (Essen Instruments) by monitoring nine regions per well every hour. The assessment of cell proliferation was based on confluence as calculated by the integrated IncuCyte software. For measurement of clonogenic cell survival, HeLa cells were plated at 3 × 10^5^/well in six-well plates. After 24 h, cells were transfected with 100 nM siRNA (Tdg-directed or control) using DharmaFECT 1 according to the manufacturer's instructions. Twenty-four hours after transfection, cells were trypsinized, counted, and then plated in duplicate in 10-cm dishes at cell densities between 200 and 800 cells/dish in growth medium containing 5-FU concentrations as indicated. Remaining cells were further cultivated in normal growth medium for Western blot analysis of TDG protein after 72 h. Seventy-two hours after plating, the medium of 5-FU and mock-treated cells was exchanged with 5-FU–free growth medium, and plates were incubated for additional 14 d. Colonies were visualized by staining with a 10% Giemsa solution after washing with PBS and fixation with 50% methanol.

### Purification of recombinant mTDGa.

For expression of mTDGa, 2 l of Superbroth containing 50 μg/ml kanamycin were inoculated with an overnight culture of Escherichia coli BL21(DE3) transformed with pET-28c-m*TdgA*. The culture was grown to an A_600_ (absorbance at 600 nm) of 0.6 and cooled to 15 °C. TDG expression was induced by the addition of 200 μM IPTG and incubation was allowed to proceed at 15 °C for 23 h. Cells were collected by centrifugation (Sorvall SLC-6000, 5,000 rpm, 4 °C, 30 min), and the pellets were resuspended in 3 ml/g sonication buffer (50 mM Na-phosphate [pH 8.0], 750 mM NaCl, 20% glycerol, 1 mM imidazole, 10 mM β-mercaptoethanol, 1 mM phenylmethylsulfonyl fluoride). After shock freezing in liquid nitrogen, cells were stored at −80 °C. Crude extracts were prepared by sonication (12 times for 30 s on ice with intermittent chilling), and clarified by centrifugation (Sorvall SS34, 18,000 rpm, 4° C). All steps were performed at 4 °C. The supernatant was applied to a disposable column packed with 1.5 ml of pre-equilibrated Ni-NTA agarose (Qiagen) at a flow rate of 15 ml/h. After washing with 120 ml of buffer, bound proteins were eluted with 10 ml of sonication buffer containing 500 mM imidazole and dialyzed against buffer H50 (50 mM Na-phosphate [pH 8.0], 50 mM NaCl, 20% glycerol, 10 mM β-mercaptoethanol, 1 mM phenylmethylsulfonyl fluoride). After loading the dialyzed fraction onto a 5-ml HiTrap Heparin HP column (GE Healthcare) at a flow rate of 1 ml/min and washing with 10 ml of H50, bound protein was eluted with a liner gradient of 50–800 mM NaCl in 50 ml. Purest fractions were pooled, dialyzed against buffer Q20 (50 mM Na-phosphate [pH 8.5], 20 mM NaCl, 10% glycerol, 10 mM β-mercaptoethanol, 1 mM phenylmethylsulfonyl fluoride), and loaded on a 1-ml HiTrap Q HP at a flow rate of 1 ml/min. After washing with 10 ml of Q20 buffer, bound proteins were eluted with a linear gradient of 20–500 mM NaCl in 15 ml. The fractions containing TDG with >98% homogeneity were pooled, dialyzed against storage buffer (50 mM Na-phosphate [pH 8.0], 50 mM NaCl, 10% glycerol, 10 mM β-mercaptoethanol, 1 mM phenylmethylsulfonyl fluoride), frozen in liquid nitrogen and stored at −80 °C.

### Base release assays and assessment of genomic uracil and 5-FU.

Nuclear protein extracts were prepared according to [33]. For base release assays, 20 μg of nuclear proteins were incubated with 1 pmol of a fluorescein-labeled homoduplex or mismatched DNA substrate (2) in reaction buffer (50 mM Tris-HCl [pH 8.0], 1 mM EDTA, 1 mM DTT, 1 mg/ml BSA, 2 U UGI) for 20 h at 37 °C. Generated AP-sites were cleaved by the addition of NaOH to a final concentration of 100 mM and heating to 95 °C for 10 min. Subsequently, DNA was ethanol precipitated overnight at −20 °C in 0.3 M Na-acetate (pH 5.2) and in the presence of 0.4 mg/ml carrier tRNA. The DNA was collected by centrifugation (20 min, 20,000*g*, 4 °C) and washed in 80% ethanol. Air-dried pellets were resuspended in loading buffer (1× TBE, 90% formamide), heated at 95 °C for 5 min, and then immediately chilled on ice. Reaction products were separated on 15% denaturing polyacrylamide gels in 1× TBE. The fluorescein-labeled DNA was visualized with a Typhoon 9400 (GE Healthcare) and quantified using the ImageQuant TL software (GE Healthcare). Levels of genomic uracil and 5-FU were determined as described in [9] after treatment of cells with 10 μM 5-FU for 24 h.

### Alkaline Comet assays.

Comet assays were performed according to [50] with minor modifications described by [51]. After treatment, cells were trypsinized, collected by centrifugation (5 min, 450*g*, RT), and then washed with PBS. Ten thousand cells were resuspended in 100 μl of low-melting-point agarose (PBS, 0.5% LMPA; Lonza) at 37 °C, and casted onto microscope slides precoated with 1.5% normal melting agarose (BioRad). After gelling, cells were lysed by immersion of the slides in freshly prepared ice-cold lysis buffer (2.5 M NaCl, 100 mM EDTA, 10 mM Tris/HCl [pH 10], 1% TritonX-100, 10% DMSO) for 90 min at 4 °C. Slides were then washed with ddH_2_O and covered with fresh electrophoresis buffer in an electrophoresis tank (1 mM EDTA, 300 mM NaOH [pH >13]). After DNA denaturation for 30 min at 4 °C, electrophoresis was performed at 25 V and 300 mA for 20 min. All the above steps were done under dimmed light. Neutralization was carried out by three washings of 10 min with 0.4 M Tris/HCl (pH 7.5) at RT. After two fixation steps of 5 min in 100% EtOH at RT, slides were air-dried and stained with 50 μl PI solution (Vectashield, 2.5 μg/ml propidium iodide). Comet tail moments of 100 to 150 cells per slide were analyzed by automated analysis [52] using a Leitz MIAS image analyzer (Leitz Messtechnik) together with a Leica DM RBE microscope (Leica Microsystems).

### FACS analysis.

A total of 5 × 10^5^ to 5 × 10^6^ cells were fixed overnight in 5 ml of 70% ethanol at 4 °C, collected by centrifugation (5 min, 800*g*, 4 °C) and resuspended in 0.3 ml of RNase solution (100 mM Tris/HCl [pH 7.5], 100 mM NaCl, RNase 0.5 mg/ml). RNA digestion was performed at 37 °C for 45 min; 0.3 ml of PE solution (0.4% HCl, pepsin 1 mg/ml) was added to the samples during the last 15 min of incubation. The DNA was stained by the addition of 0.6 ml PI solution (PBS, 50 μg/ml propidium iodide) on ice for 30 min. Samples were analyzed by flow cytometry with a FACS Canto ll cytometer (Beckton Dickinson). Cell-cycle distribution was analyzed using the FlowJo software (TreeStar).

### Immunofluorescence.

MEFs were cultivated on coverslips for 24 h. Cells were then treated with 5 μM 5-FU for 24 h followed by cultivation in drug-free medium for another 24 h. Coverslips were then washed twice in PBS and the cells fixed for 15 min with PF-buffer (PBS, 2% paraformaldehyde) at RT, washed 4× 10 min in PBS at RT, and permeabilized in ice-cold P-buffer (PBS, 0.2% TritonX100) for 5 min. Coverslips were incubated for another 5 min in ice-cold P-buffer containing 0.2% NaBH_4_. After blocking twice in H-buffer for 10 min (PBS, 1% BSA) and once in D-buffer for 10 min (PBS, 1:20 donkey serum), samples were hybridized with the anti-XRCC1 (1:100 dilution in H-buffer) or the anti-γH2AX (1:500 dilution in H-buffer) antibody for 1 h at RT. Following four washes of 10 min in H-buffer, the samples were hybridized with the respective Cy2-conjugated secondary antibody at a dilution of 1:500 (XRCC1) or 1:200 (γH2AX) in H-buffer for 1 h at RT. After four washes of 10 min in PBS, the coverslips were dried and embedded in Mowiol (Calbiochem, Germany). XRCC1 or γH2AX signals were visualized on an Axiovert 200M microscope (Zeiss) using a FITC filter (excitation 492 nm, emission 520 nm).

### Statistical analysis.

Statistical analyses were done using the Prism 5 software (GraphPad Software). Comet tail moment data were analyzed according to P. Duez et al., 2003 [53] by two-way ANOVA of medians and 75% percentiles obtained from three independent experiments, followed by the Bonferroni post test. XRCC1 and γH2AX foci distributions were tested for normality by the Shapiro-Wilk test and further evaluated by one-way ANOVA using the nonparametric Kruskal-Wallis test followed by the Dunn multiple comparison analysis comparing treated with untreated samples. Analysis of cell-cycle data was done by the Fisher exact test from contingency tables comparing the distributions of G1-, S-, and G2-phase cells.

## Supporting Information

Figure S1 Gene Targeting of m*Tdg* on Chromosome 10 The targeting construct was generated by replacement of exons 6 and 7 with a neomycin resistance cassette. This was achieved by the substitution of a NarI-PacI fragment with the resistance cassette in a subcloned genomic region spanning from intron 4 to the 3′ UTR. In addition, the targeting construct contained a thymidine kinase (TK) cassette for negative selection. Arrowheads show the position of primers used for multiplex-PCR genotyping. The expected length of PCR products are 1.2 kb and 1.5 kb for the wild-type (wt) and the targeted loci, respectively. The lower panel shows a typical result from genotyping of wt (+/+), heterozygous (+/−), and null (−/−) Tdg MEF.
(619 KB TIF)

Figure S2 MEFs Heterozygous for TDG Display an Intermediate Fu Sensitivity The sensitivity of TDG^+/+^, TDG^+/−^, and TDG^−/−^cell lines to increasing amounts of 5-FU was measured after a continuous treatment of 48 h. The panel shows cell survival as percentage of untreated cells averaged from three independent experiments. When compared to wt MEF, cells carrying a homozygous disruption of the TDG gene were resistant to 5-FU treatment. The sensitivity of a cell line with heterozygous TDG genotype was in between the sensitivities measured for wt and knockout MEF. Error bars represent standard deviations.
(240 KB TIF)

## Abbreviations

5-FU: 5-fluorouracil
AP-site: apyrimidinic/apurinic site
ES: embryonic stem
FdUMP: fluorodeoxyuridine monophosphate
HU: hydroxyurea
MEF: mouse embryonic fibroblast
MMR: mismatch repair
MMS: methyl methansulfonate
siRNA: small interfering RNA
SSB: single-stranded DNA break
Smug1: single-strand–selective monofunctional uracil-DNA glycosylase 1
TDG: thymine DNA glycosylase
TS: thymidylate synthase
UDG: uracil DNA glycosylase
